# Complex and multilevel left ventricular outflow tract obstruction: What can 3D echocardiography add?

**DOI:** 10.1186/s43044-021-00197-y

**Published:** 2021-08-26

**Authors:** Hoda Abdelgawad, Mahmoud Shehata, Mahmoud Abdelnabi, Abdallah Almaghraby, Mohamed Ayman Abdel-Hay

**Affiliations:** 1 Cardiology Department, Faculty of Medicine, Alexandria University, Champollion Street, Khartoom Square, Qism Bab Sharqi, Alexandria, Egypt; 2 Cadiology Department, Faculty of Medicine, Port Said University, Port Said, Egypt; 3 Cardiology and Angiology Unit, Clinical and Experimental Internal Medicine Department, Medical Research Institute, Alexandria University, Alexandria, Egypt; 4 Internal Medicine Department, Texas Tech Univeristy Health Science Center, Lubbock, Texas USA

**Keywords:** 3D echocardiography, Subaortic membrane, Aortic valve stenosis, Left ventricular outflow tract obstruction

## Abstract

**Background:**

Subaortic obstruction by a membrane or systolic anterior motion of the mitral valve leaflets is usually suspected in young patients, especially if the anatomy of the aortic valve is not clearly stenotic and unexplained left ventricular hypertrophy exists in the context of high transaortic gradients.

**Main body:**

In certain circumstances, some patients show both aortic and subaortic stenotic lesions of variable severity. Doppler echocardiography can help in grading severity in the case of single-level obstruction but not in patients with multilevel obstruction where the continuity equation is of no value. Three-dimensional (3D) echocardiography allows "en-face" visualization of each level of the aortic valve and subaortic tract; in addition, direct planimetry of the areas can be done using multiplanar reformatting.

**Conclusions:**

Accordingly, 3D echocardiography plays a crucial role in the assessment in patients with multilevel left ventricular outflow tract obstruction as it can accurately delineate the location and size, and severity of the stenosis.

**Supplementary Information:**

The online version contains supplementary material available at 10.1186/s43044-021-00197-y.

## Background

Obstruction of the left ventricular tract usually has variable etiologies and severity. It can be subaortic, aortic, or supra-aortic stenosis, single or multilevel stenosis [1–3]. The continuity equation is the most sensitive method in grading isolated aortic valve stenosis, provided that neither significant aortic valve regurgitation nor subaortic obstruction is present [4, 5]. Subvalvular aortic stenosis may have a dynamic component and may be due to a fibrous membrane, muscular obstruction, or combined.

## Diagnosis of aortic valve stenosis in special situations

Rheumatic valve infection is endemic in some of the developing countries affecting 10:1,000–15:1,000 patients, being the commonest cause of valve surgeries and causing nearly 300,000 deaths/year. Although it commonly affects the mitral valve, aortic valve can be affected in up to 30% of cases, resulting in serious effects on left ventricular function, quality of life, and overall prognosis. Nine percent of the patients have isolated aortic stenosis, 14% have isolated regurgitation, and 6% have mixed lesions. Rheumatic post-inflammatory lesions result in thickness, fibrosis of the aortic valve, and shrinkage of the cusps usually with fusion at the commissures and sometimes calcification [6–8].

Like the rheumatic mitral valve, the aortic valve shows systolic doming that makes proper planimetry of the narrowest orifice difficult to be done by the conventional two-dimensional (2D) transesophageal echocardiography (TOE). Moreover, coexisting mitral valve stenosis is commonly encountered. This can lead to paradoxically low gradients across the aortic valve [9–12].

3D echocardiography shows greater sensitivity in comparison with 2D TOE, especially with the use of multiplanar reformatting (MPR) of the 3D volumes and biplane mode. With the help of MPR, 3D-derived LVOT and aortic valve areas can be accurately traced.

## Localization of the level of obstruction

Conventional 2D echocardiography allows the localization of the obstruction using Doppler echocardiography yet, it is not useful in patients with severe aortic valve regurgitation and in combined valvular and subvalvular stenosis where continuity equation will not be valid.

## Proposed protocol

With the advent of 3D echocardiography, planimetered areas of the aortic valve and LVOT can be easily traced.

It is recommended to start with 2D parasternal views with color Doppler to appreciate the aortic valve opening, presence of subaortic membrane and associated systolic anterior motion (SAM) of the mitral valve leaflets.

**Fig. 1:**
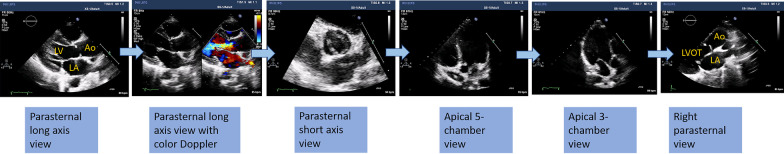
2D TTE imaging sequence for the aortic–subaortic complex

Apical 5- and 3-chamber views and right parasternal view if possible are of great value to measure the gradients across the LVOT (Fig. 1, Additional file 1: Video S1).

**Fig. 2:**
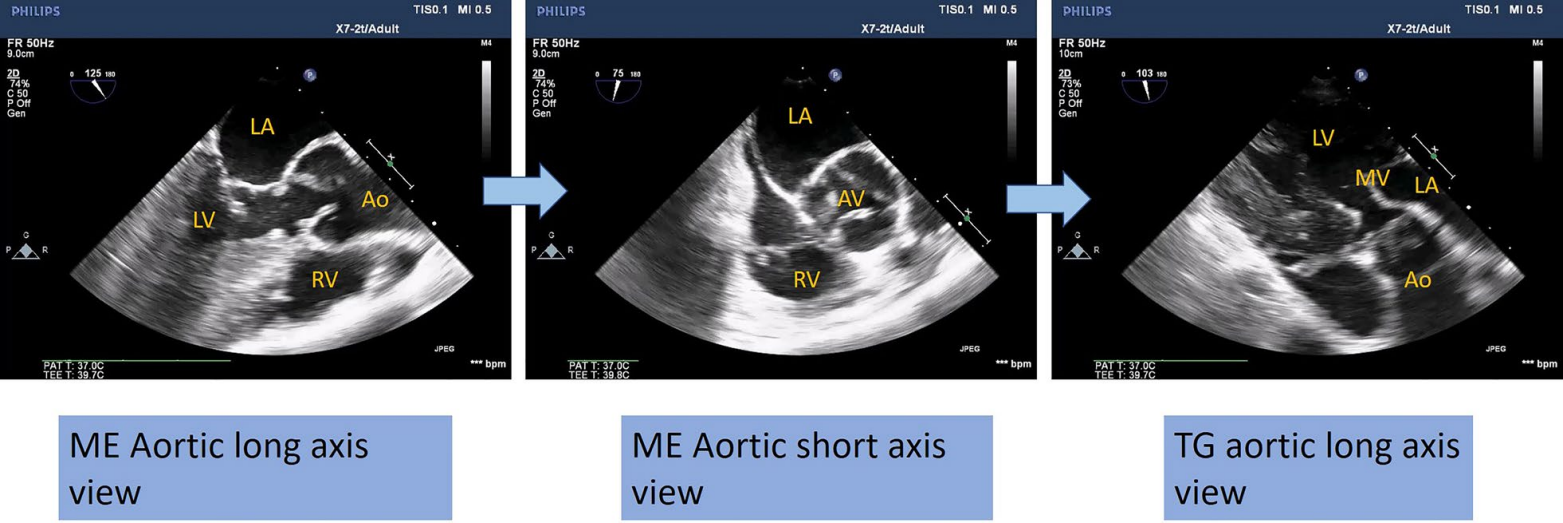
2D TOE imaging sequence for the aortic–subaortic complex

Using 2D TOE, aortic long- and short-axis mid-esophageal views and aortic transgastric views are more sensitive than transthoracic echocardiography (TTE) to clearly assess the above-mentioned data (Fig. 2).

**Fig. 3:**
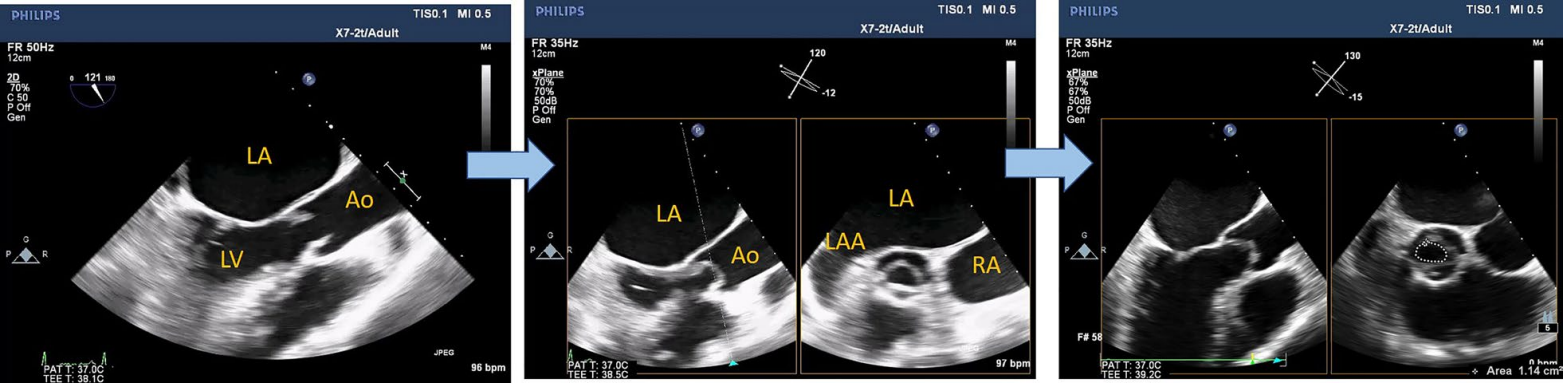
X-plane-derived aortic valve area

**Fig. 4:**
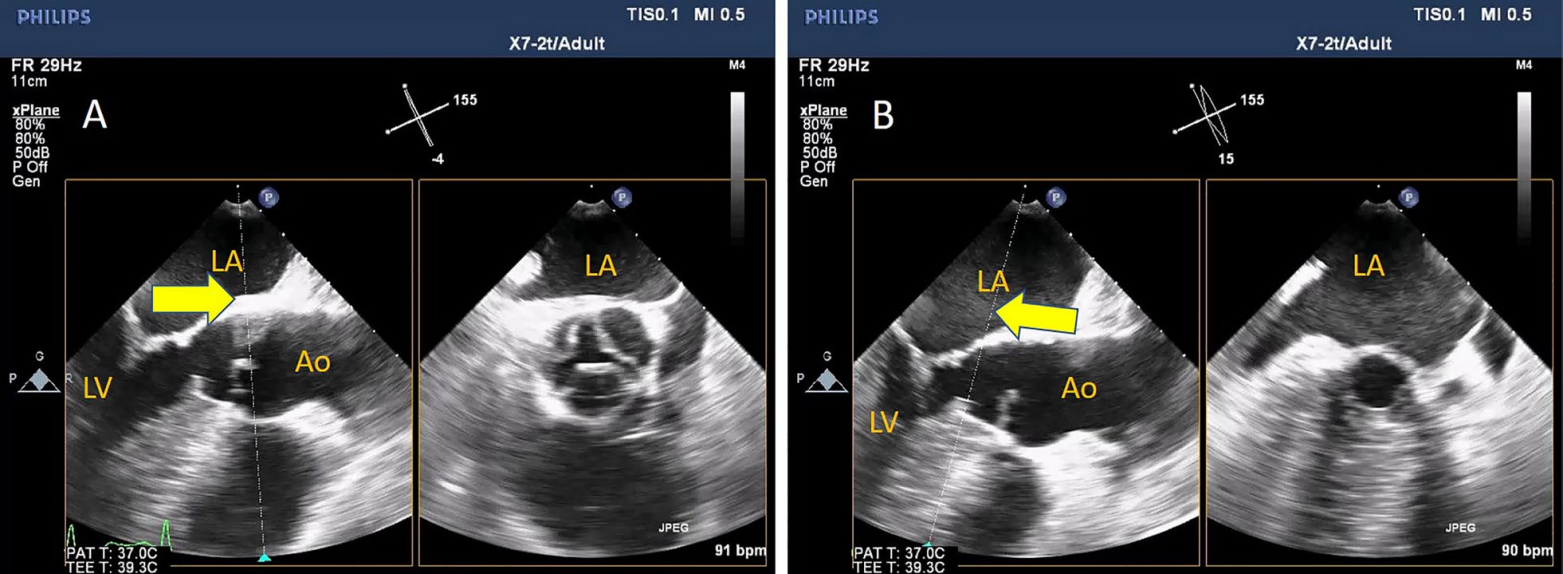
X-plane-derived aortic valve area (panel A) and subaortic area at the level of the subaortic membrane (panel B)

3D-derived X-plane, zoomed and full-volume multi-beat modes of acquisition are highly recommended to grade the severity of the stenosis. In the X-plane mode, TOE-derived aortic long-axis is obtained (nearly at ˜120 ˚), the line at the reference clip should be placed at the level of obstruction (Aortic cusps' tips or subaortic membrane) and then frozen the clip at the mid-systolic frame, and then, either the aortic valve area (AVA) or LVOT area can be easily traced in the corresponding perpendicular plane (Figs. 3, 4, Additional file 2 and 3: Video S2, S3).

**Fig. 5:**
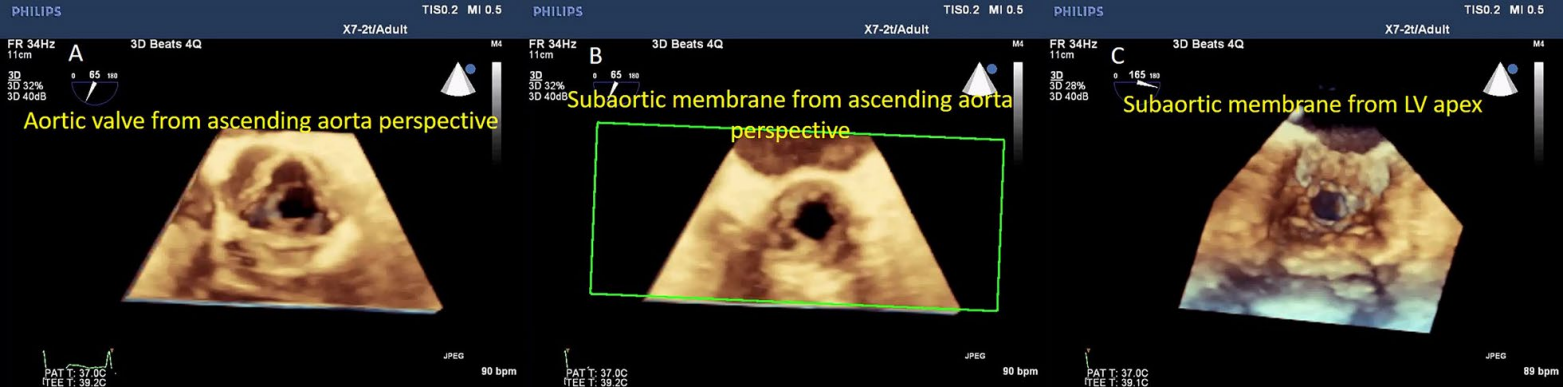
3D zoomed mode, "en-face" view of the aortic valve (panel A) and subaortic membrane from the ascending aorta perspective (panel B) and subaortic membrane from LV apex (panel C)

Using zoomed and full-volume multi-beat modes, "en-face" views of the aortic and subaortic planes can be easily obtained. The morphology and dynamic variations of each level are much easier and faster to be assessed in comparison with 2D TOE (Fig. 5, Additional file 4, 5 and 6: Video S4–S6).

Moreover, with routine exercising of MPR-derived measurements, areas of the valves and stenotic tracts can be easily traced as they are less susceptible to gain artifacts (Figs. 6, 7, 8, 9, 10, 11, 12, 13, 14, Additional file 7 and 8: Video S7, S8). A stepwise approach for the assessment of aortic–mitral and aortic–subaortic stenoses is illustrated in Fig. 15.

**Fig. 6:**
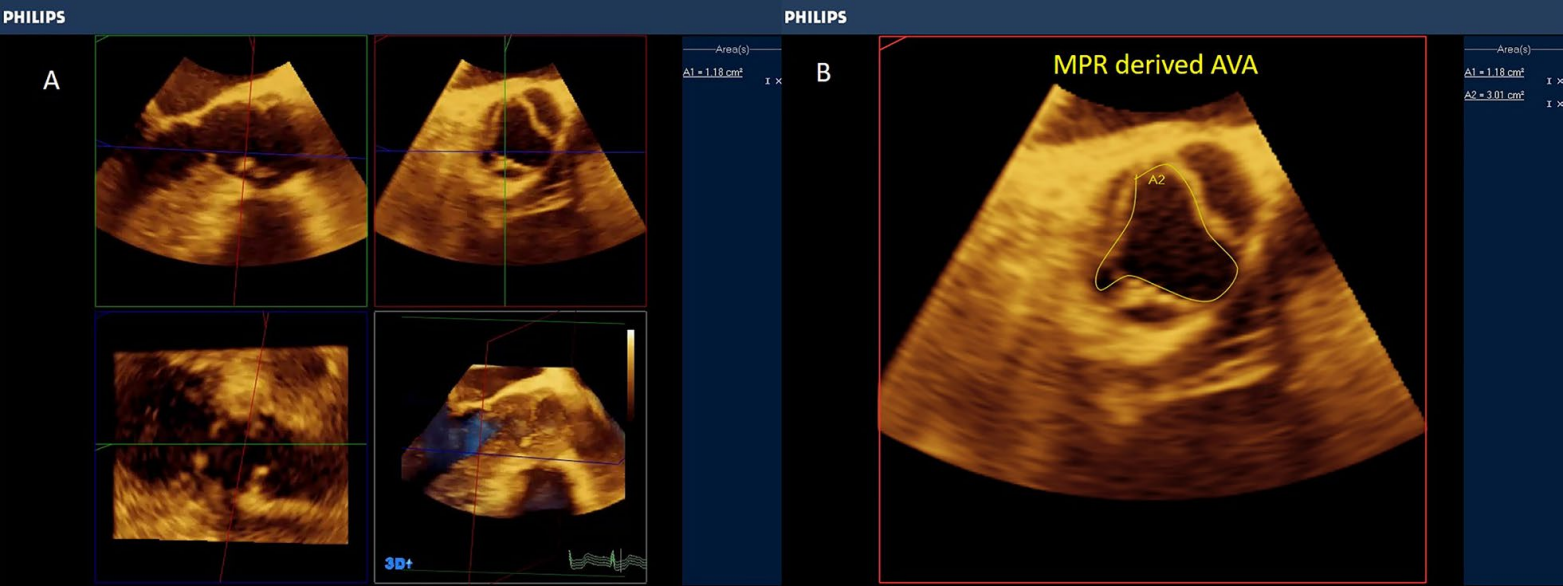
Multiplanar reformatting (MPR) of the 3D volume of the aortic valve. (panel A) Direct planimetry of the narrowest AV orifice was obtained (Panel B)

**Fig. 7:**
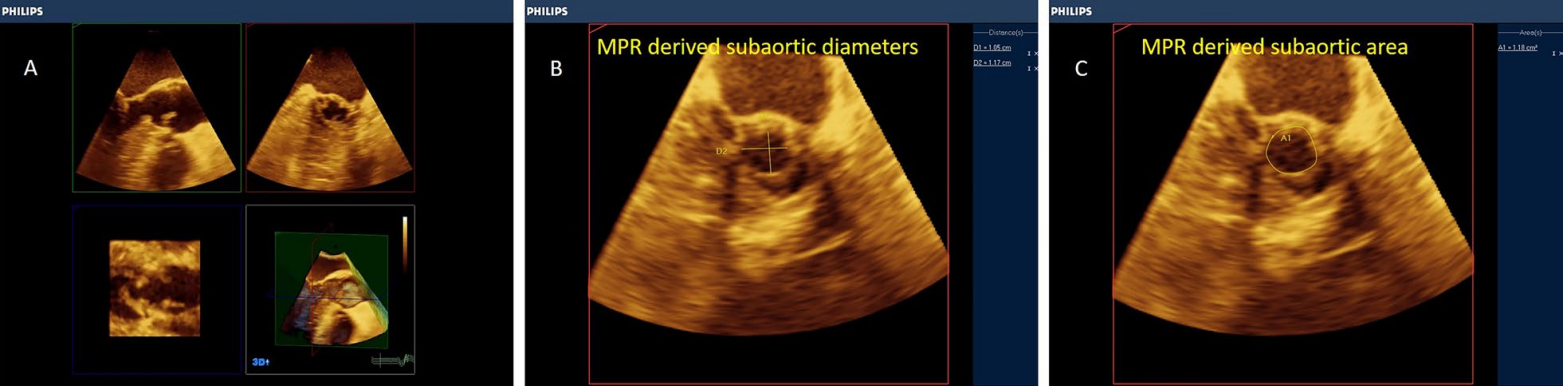
Multiplanar reformatting (MPR) of the 3D volume of a subaortic membrane. (panel **A**) Maximum diameters and planimetered area of the LVOT at the subaortic membrane (Panel **B**, **C**)

**Fig. 8:**
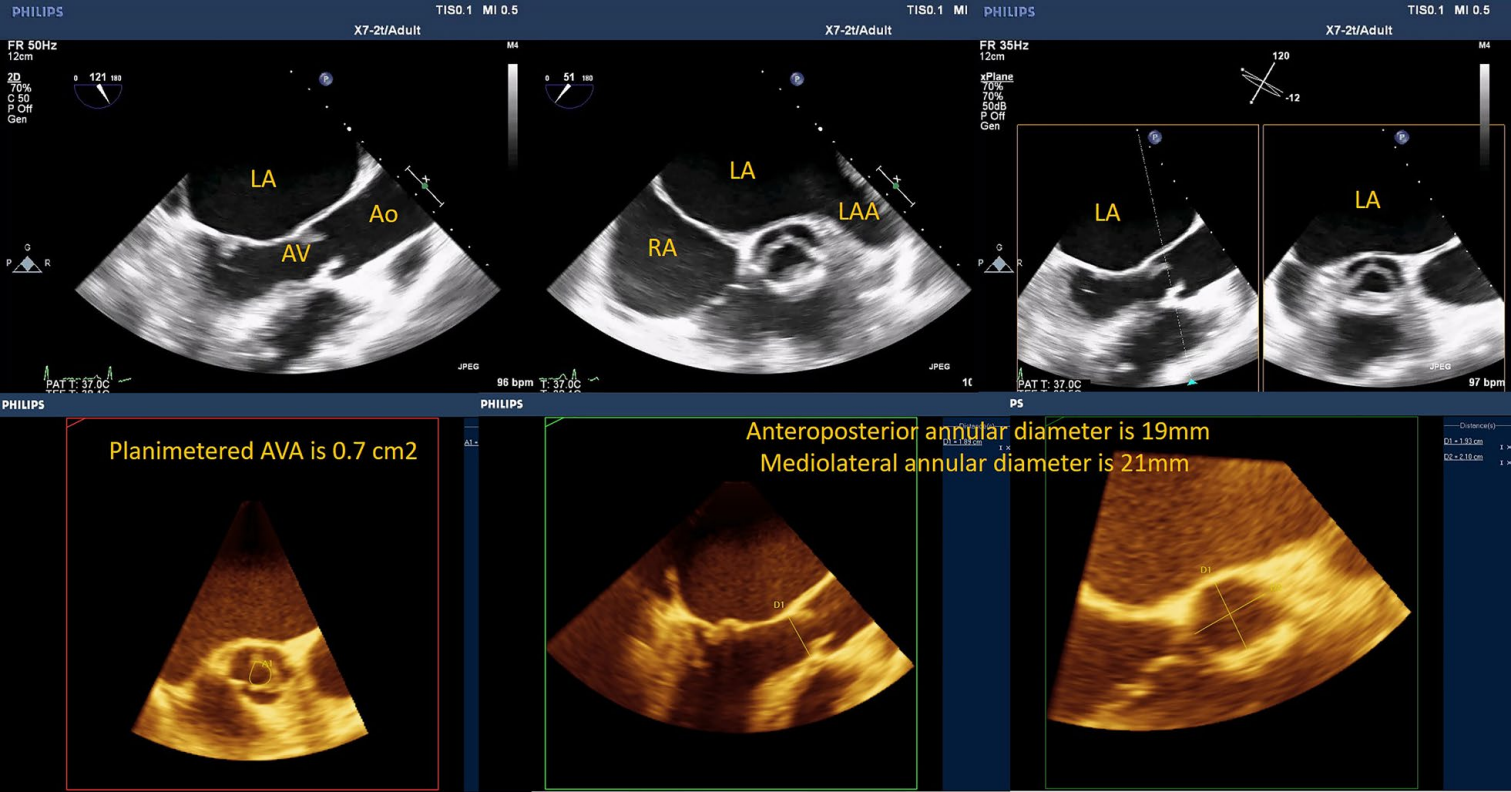
A case of rheumatic aortic valve disease. 2D TOE (aortic long- and short-axis views) shows reduced excursion of the cusps with characteristic doming. X-plane mode of the aortic valve at the level of its tips to get the true anatomical orifice. Multiplanar reformatting (MPR) of the 3D volume of the aortic valve and LVOT; accurate planimetered AVA and the anteroposterior and mediolateral diameters of the aortic annulus

**Fig. 9:**
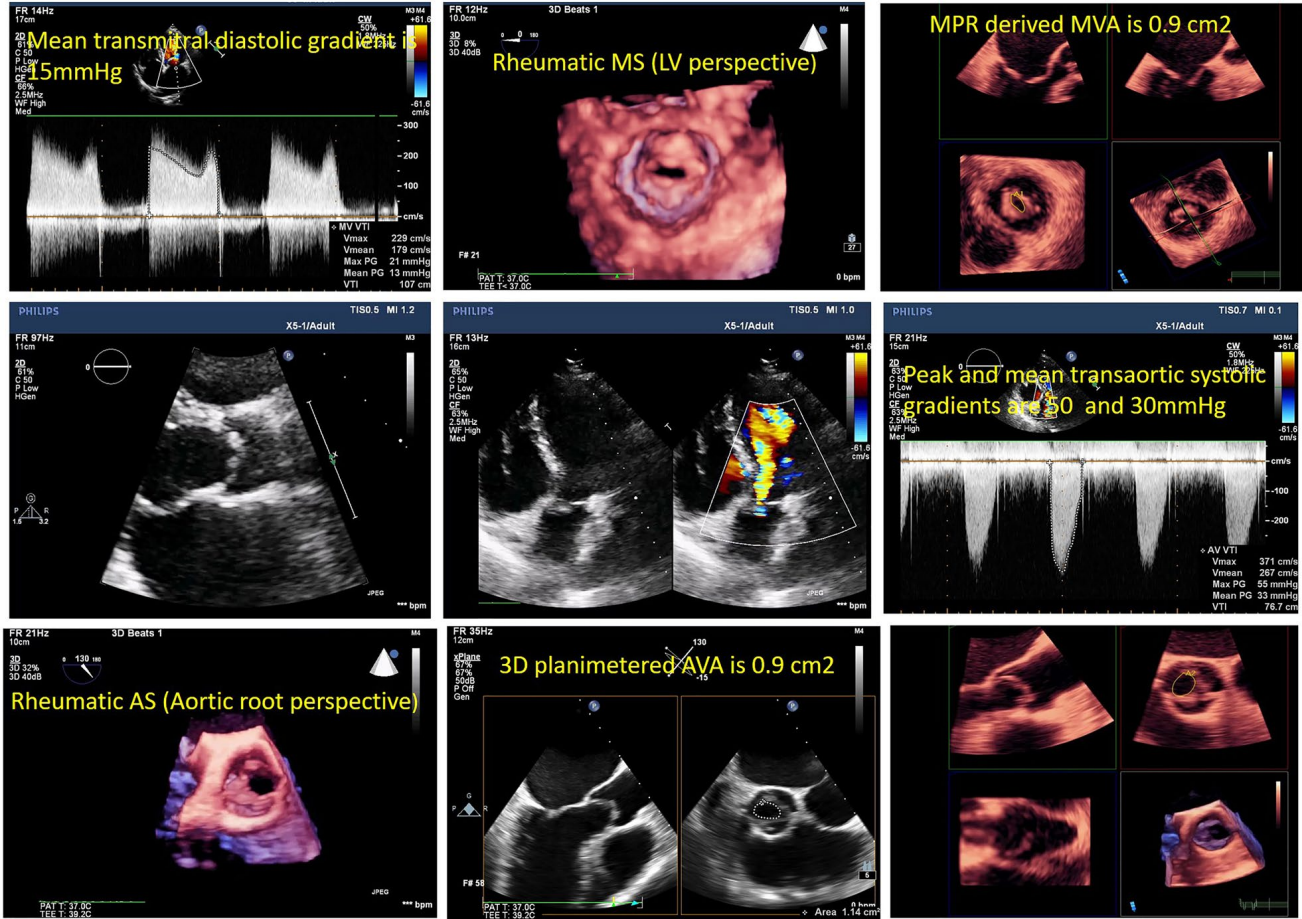
A case of rheumatic mitral and aortic valve diseases. In the upper panel, severe mitral valve stenosis was found; mean diastolic gradient = 15 mmHg .3D zoomed volume shows the characteristic "en-face" view of the thickened leaflets and commissural fusion. MVA was traced using MPR as 0.9 cm2. In the middle panel, a 2D parasternal long-axis view of the aortic valve shows systolic doming of the cusps. The apical 5-chamber view shows aortic valve regurgitation. In the lower panel, 3D-zoomed volume of the aortic valve shows thickened cusps and fused commissure between right and non-coronary cusps. X-plane mode and MPR-derived AVA are 0.9 cm2

**Fig. 10:**
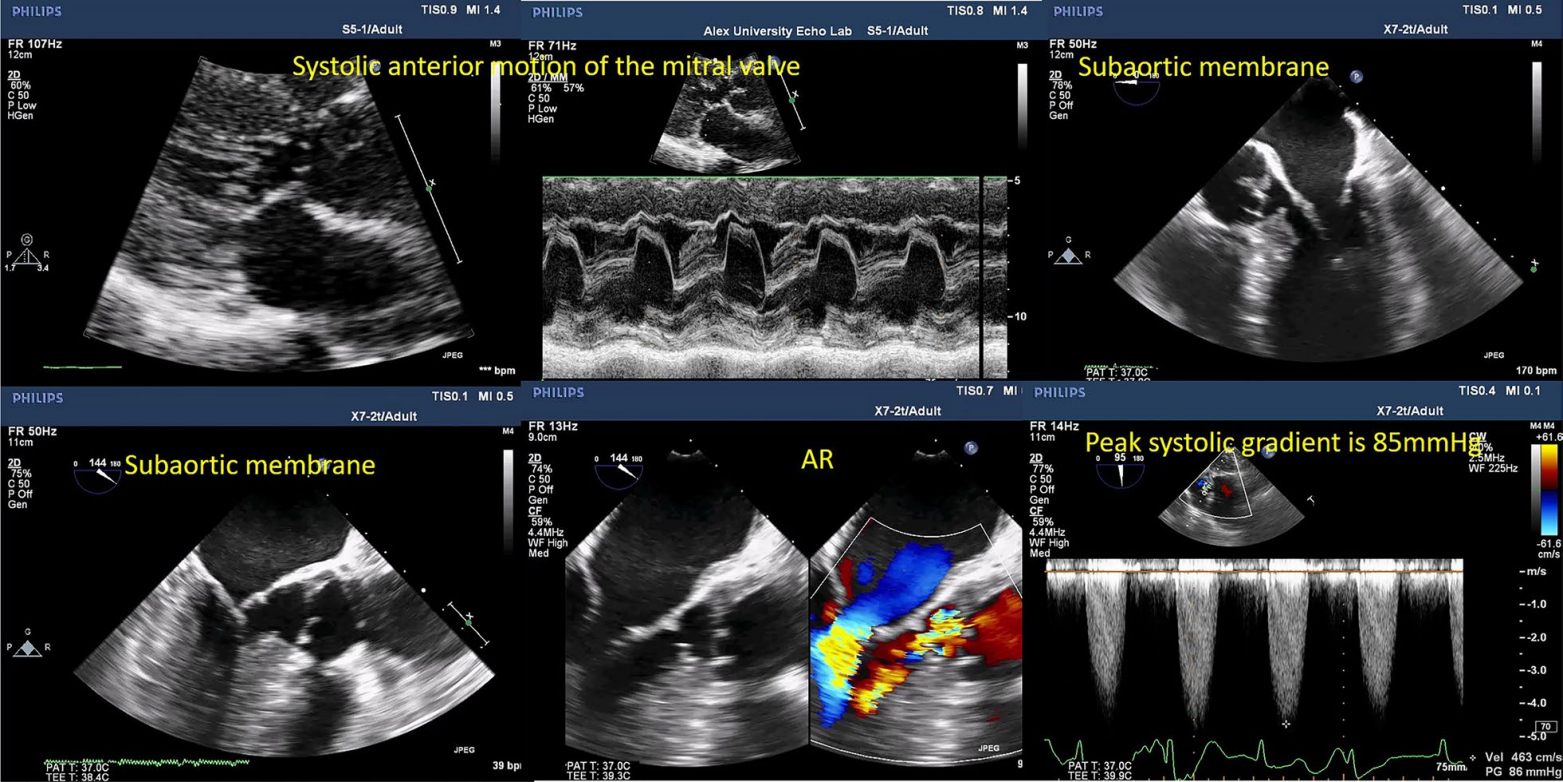
A right upper and middle panels, parasternal long-axis view, and M-mode show left ventricular hypertrophy and systolic anterior motion of the mitral valve. 2D TOE (left upper and right lower and middle panels) shows a subaortic membrane and aortic valve regurgitation. In the left lower panel, high peak and mean transaortic gradients

**Fig. 11:**
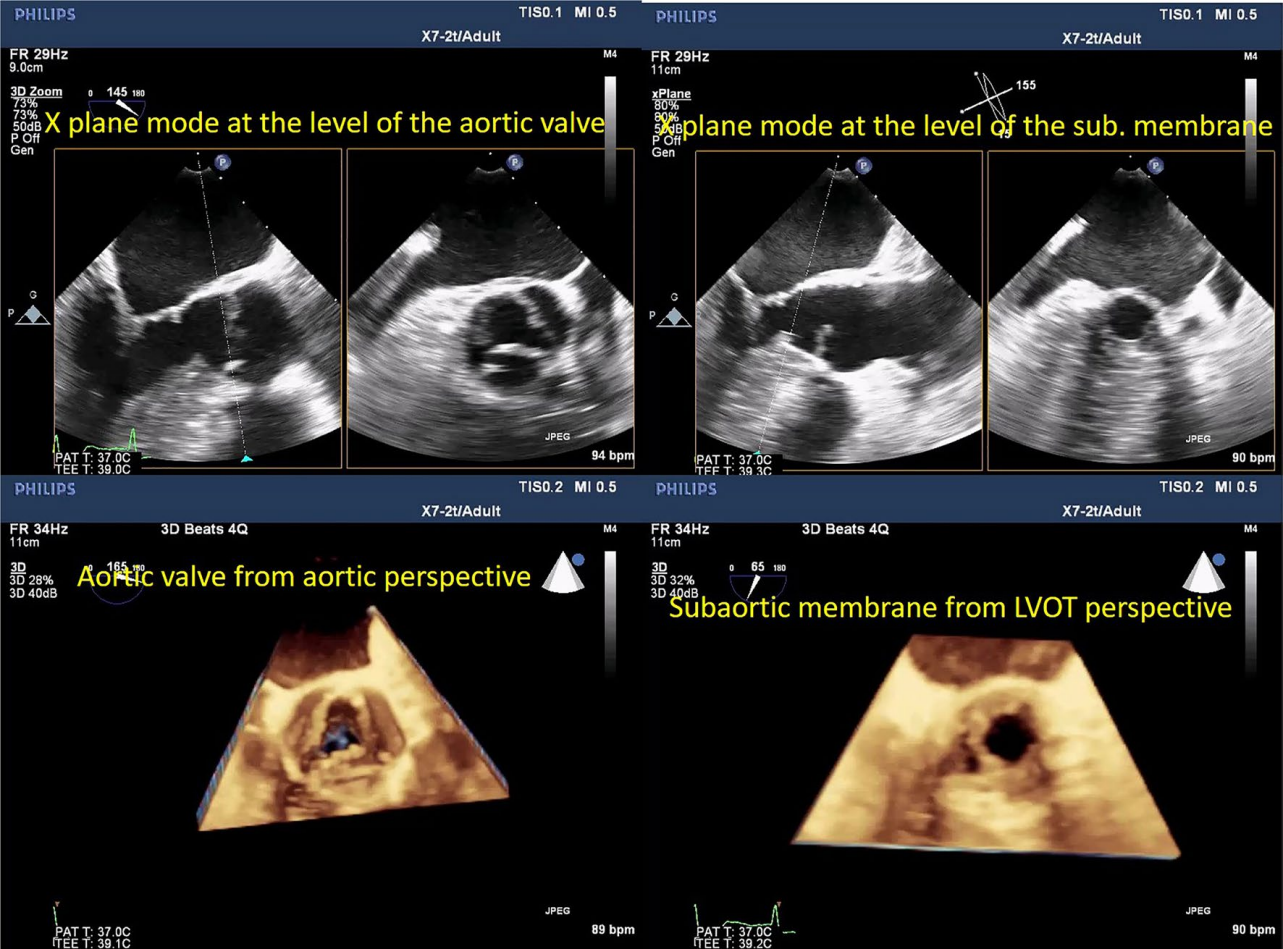
Right upper panel, X-plane mode of the aortic valve, the anatomical orifice of the aortic valve is seen in the perpendicular image. Left upper panel, X-plane mode of the subaortic membrane (moving the reference line slightly to the right), the functional opening of the LVOT at the level of the subaortic membrane is clearly seen. Right lower panel, 3D-zoomed volume of the aortic valve from the ascending aorta perspective shows the trileaflet valve with thickened cusps. Left lower panel, 3D-zoomed volume (after cropping of the aortic valve plane) shows a circumferential muscular membrane with a circular opening

**Fig. 12:**
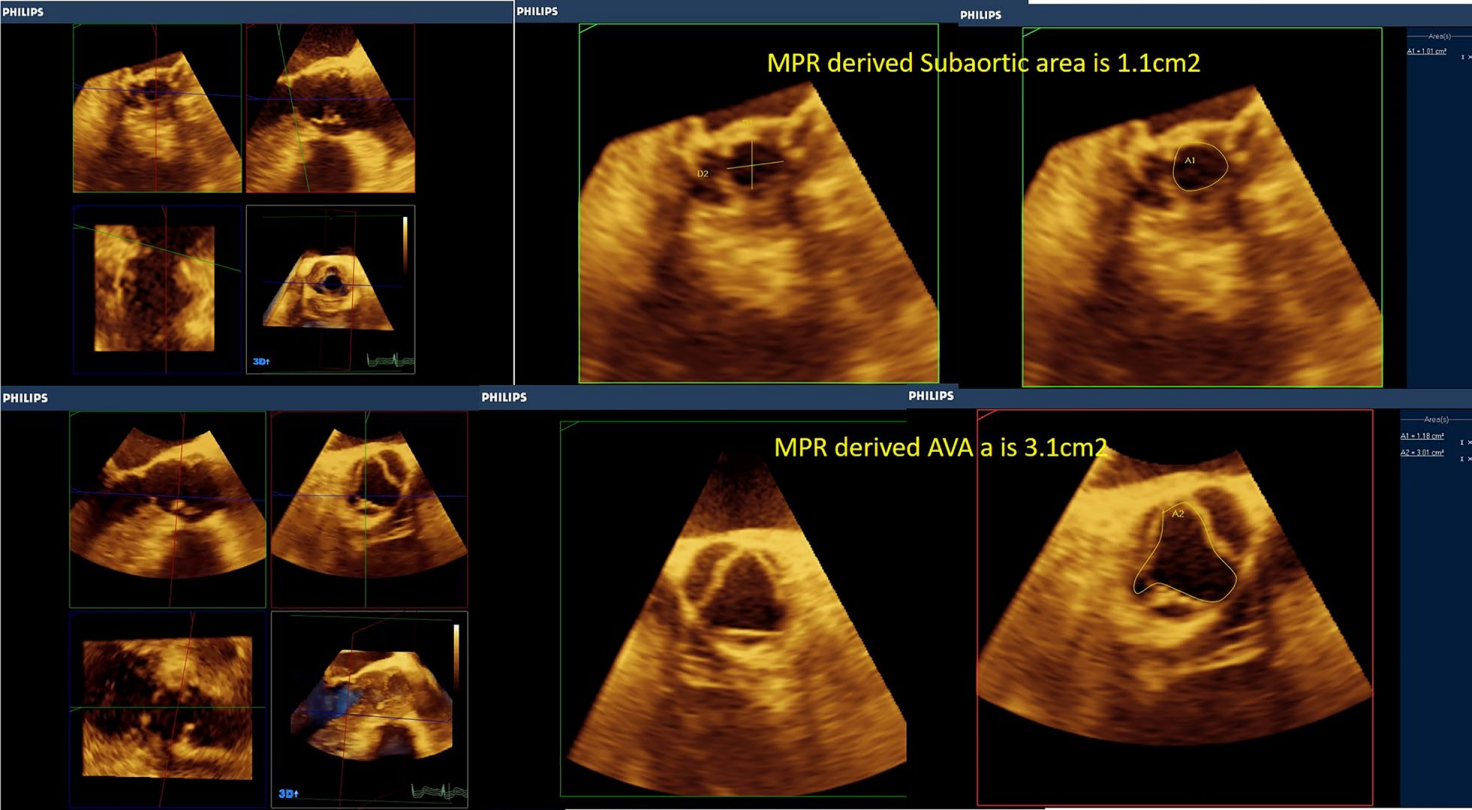
Upper panels, multiplanar reformatting (MPR)-derived diameters and area of the LVOT at the level of the subaortic membrane. (10 X 12 mm and 1.1 cm2, respectively) Lower panels, multiplanar reformatting (MPR)-derived aortic valve area (mid-systolic and at the tips) is 3.1 cm2

**Fig. 13:**
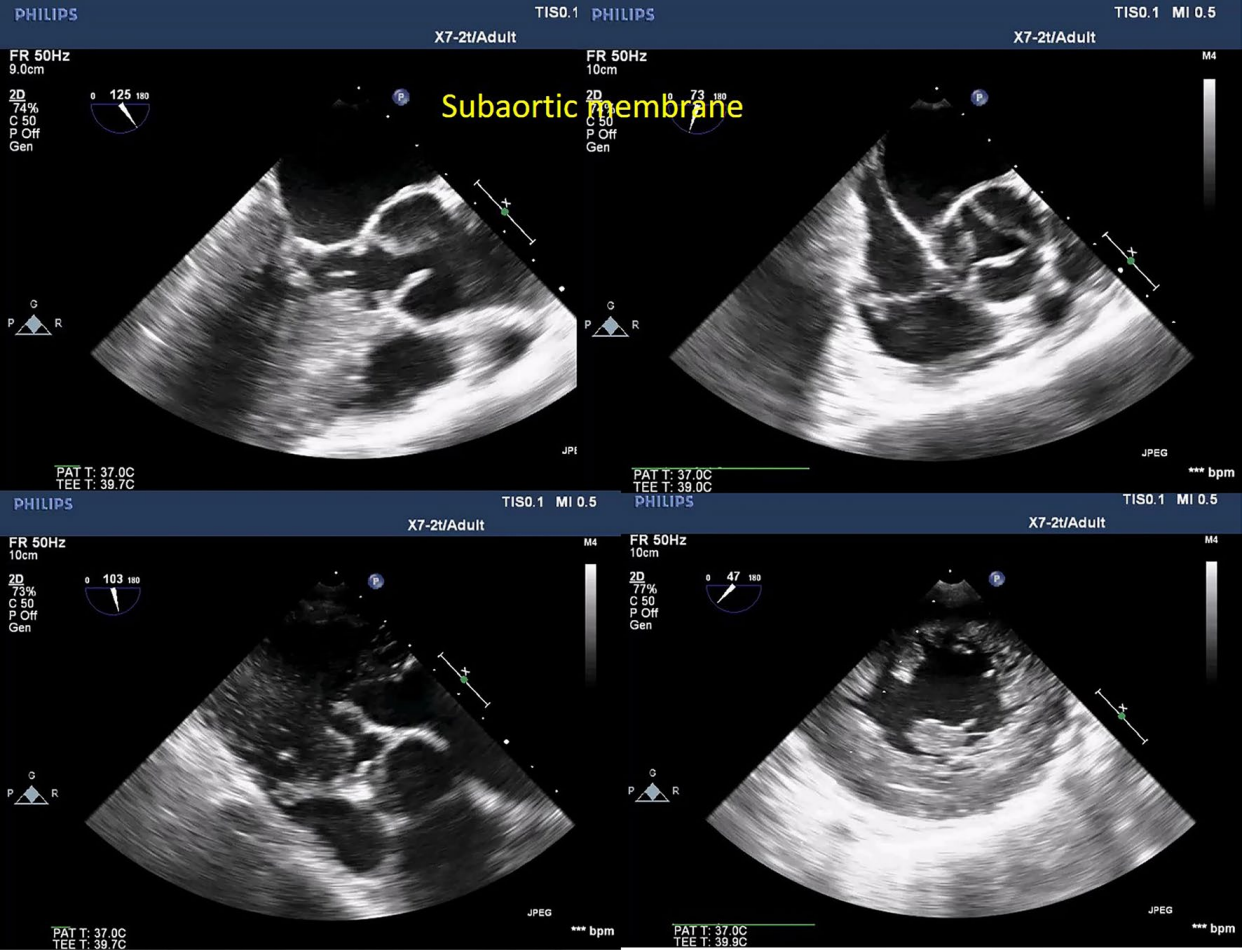
Right upper panel, 2D TOE (ME aortic long-axis view) shows small rims of the subaortic membrane seen immediately adherent to the aortic cusps. Left upper panel, 2D TOE (ME aortic short-axis view) shows the trileaflet aortic valve. Right lower panel, 2D TOE (TG aortic long-axis view) shows a small tunnel of the LVOT. Left lower panel, 2D TOE (TG short axis of the LV) shows hypertrophy of the left ventricle and papillary muscles

**Fig. 14:**
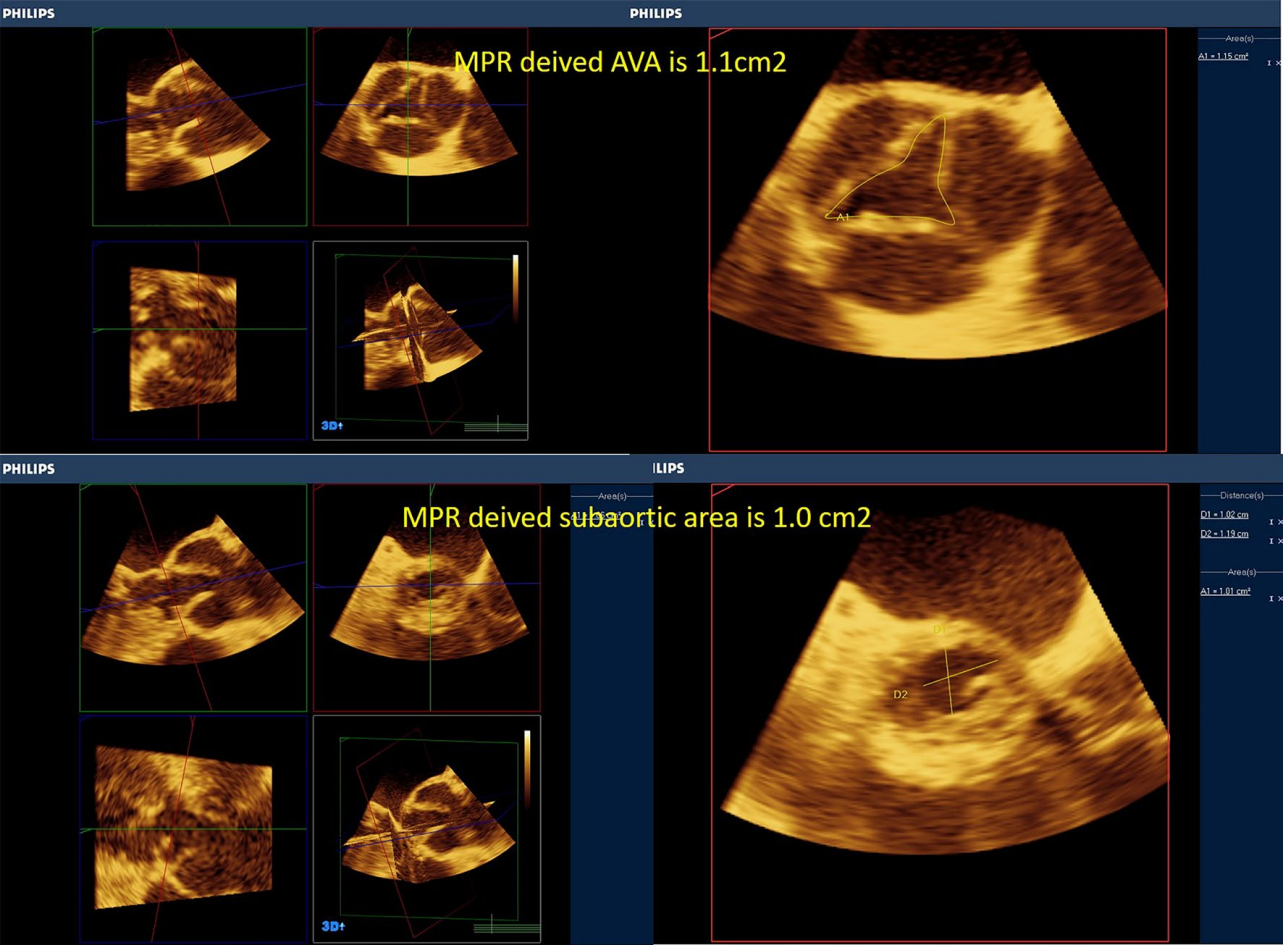
Upper panels, multiplanar reformatting (MPR)-derived aortic valve area (mid-systolic and at the tips) is 1.1 cm2. Multiplanar reformatting (MPR)-derived area of the LVOT at the level of the subaortic membrane is 1.0 cm2

**Fig. 15:**
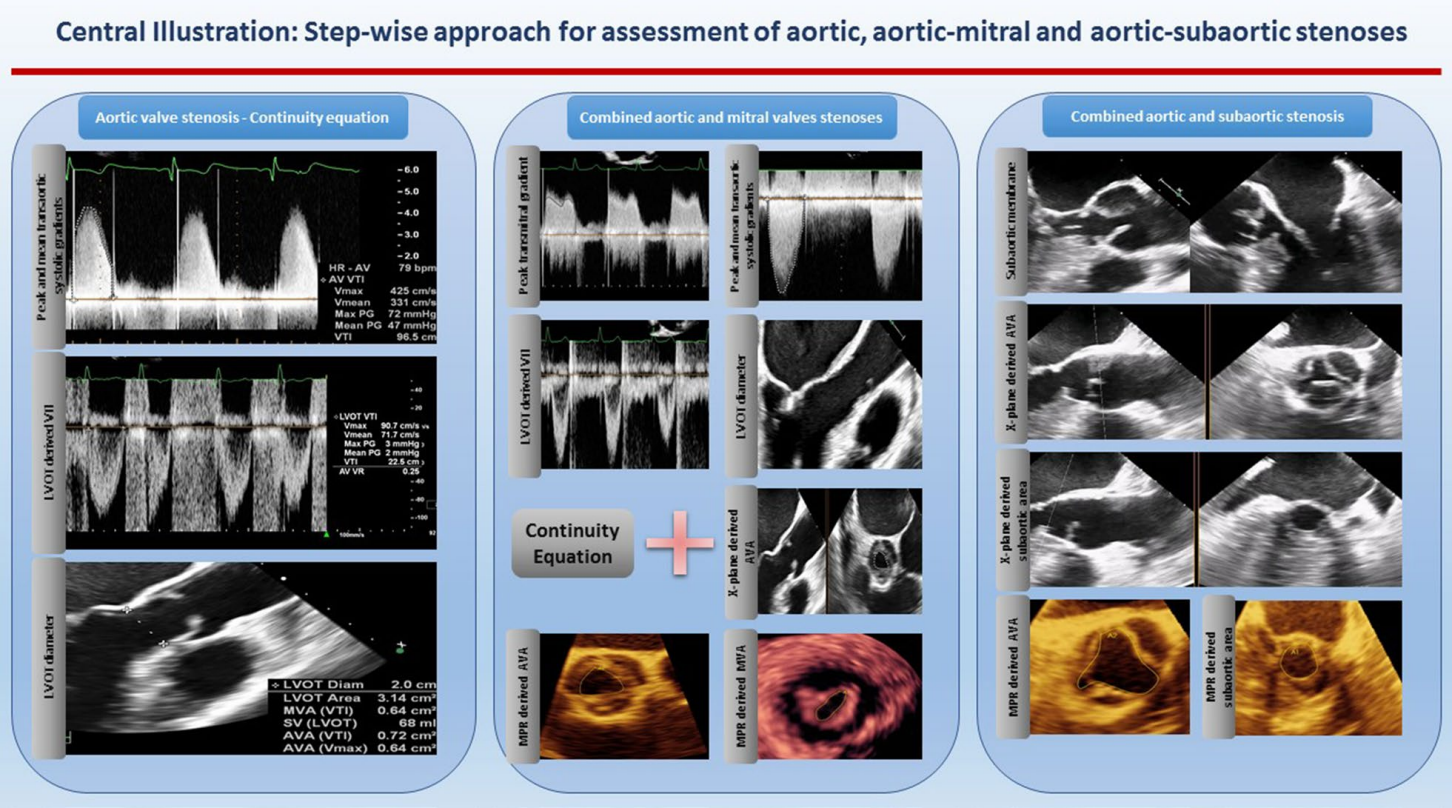
Stepwise approach for the assessment of aortic–mitral and aortic–subaortic stenoses

## Conclusions

With the revolution of 3D echocardiography, a better understanding of the anatomical details of complex lesions is no longer impossible. Being radiation-free and less invasive, 3D echocardiographic evaluation of aortic and subaortic stenosis is highly evolving with an accurate assessment of the size, location, and severity of each level of obstruction. Subsequently, 3D echocardiography is an excellent imaging tool to provide a roadmap before surgical interventions in such cases.

## Supplementary Information

**Additional file 1**. Video 1: 2D TTE, right parasternal view shows restricted and doming aortic cusps.

**Additional file 2**. Video 2: X-plane-derived aortic valve area.

**Additional file 3**. Video 3: X-plane-derived subaortic area.

**Additional file 4**. Video 4: Fluttering motion of the aortic cusps in case of the subaortic membrane.

**Additional file 5**. Video 5: Zoomed mode (LVOT perspective) shows circumferential narrowing of the LVOT with a subaortic membrane.

**Additional file 6**. Video 6: Zoomed mode (LV apex) shows a subaortic membrane.

**Additional file 7**. Video 7: Multiplanar reformatting (MPR)-derived aortic valve area.

**Additional file 8**. Video 8: Multiplanar reformatting (MPR)-derived subaortic area.

## Data Availability

The data are available for sharing.

## Abbreviations

3D: Three-dimensional
LVOT: Left ventricular outflow tract
2D: Two-dimensional
TOE: Transesophageal echocardiography
MPR: Multiplanar reformatting
SAM: Systolic anterior motion
TTE: Transthoracic echocardiography
AVA: Aortic valve area
